# Investigation on the effect of antimicrobial photodynamic therapy as an adjunct for management of deep caries lesions—study protocol for a randomized, parallel groups, controlled clinical trial

**DOI:** 10.1186/s13063-023-07181-8

**Published:** 2023-03-04

**Authors:** Luiz Filipe Barbosa Martins, Leandro Rodrigues de Sena, Diego Martins de Paula, Victor Pinheiro Feitosa, Anna Carolina Ratto Tempestini Horliana, Kristianne Porta Santos Fernandes, Raquel Agnelli Mesquita-Ferrari, Lara Jansiski Motta, Marcela Leticia Leal Gonçalves, Sandra Kalil Bussadori

**Affiliations:** 1 Post Graduation Program in Biophotonics Applied to Health Sciences, Universidade Nove de Julho, São Paulo, SP Brazil; 2 Paulo Picanço School of Dentistry, Fortaleza, CE Brazil; 3 Postgraduation Program in Health and Environment, Universidade Metropolitana de Santos, Santos, SP Brazil

**Keywords:** Photodynamic therapy, Dental caries, *Bixa orellana*, LED, aPDT

## Abstract

**Background:**

Alternatively to conventional treatments, chemo-mechanical caries removal agents can be used. A modality of treatment that has been increasing in dentistry is antimicrobial photodynamic therapy (aPDT). *Bixa orellana* is being researched for application in aPDT. This protocol aims to determine the effectiveness of aPDT with *Bixa orellana* extract in deep caries lesions.

**Methods:**

A total of 160 teeth with deep occlusal dental caries will be selected and divided into 4 groups: G1 - control group (Caries removal with a low-speed drill); G2 - Partial Caries Removal with Papacarie™ (Fórmula e Ação, São Paulo, SP, Brazil); G3 - Partial Caries Removal with Papacarie™ and application *Bixa orellana* extract (20%) (Fórmula e Ação, São Paulo, SP, Brazil); G4 - Partial Caries Removal with Papacarie™ and application *Bixa orellana* extract (20%) with LED (Valo Cordless Ultradent®, South Jordan, UT, USA) (aPDT). After treatment, all the teeth will be restored with glass ionomer cement and followed up clinically and radiographically, with evaluations at immediately, 1 week, and 1, 3, 6, and 12 months. Dentin samples before and after treatment will be analyzed microbiologically. The efficacy of treatments will be assessed with microbiological (colony-forming units, before and after carious tissue removal), radiographic (integrity of the periapical area and eventual changes in the radiolucent zones), and clinical examinations (retention of the restorative material in the cavity and occurrence of secondary caries), as well as with the time required for the procedures and the need for anesthesia during the procedures. In case data distribution is normal, analysis of variance (ANOVA) will be used for both the dependent and independent variables. In case the data distribution is not normal, the Friedman test will be used for the dependent variables. For independent variables, the Kruskal-Wallis test will be used.

**Discussion:**

Procedures using aPDT have been developed for the treatment of dental caries, but there are few controlled clinical trials in the literature confirming its efficacy.

**Trial registration:**

This protocol is registered at ClinicalTrials.gov under the number NCT05236205 and it was first posted on 01/21/2022 and last updated on 05/10/2022.

## Administrative information

Note: the numbers in curly brackets in this protocol refer to the SPIRIT checklist item numbers. The order of the items has been modified to group similar items (see http://www.equator-network.org/reporting-guidelines/spirit-2013-statement-defining-standard-protocol-items-for-clinical-trials/).

**Table:** 

Title {1}	Investigation on the effect of antimicrobial photodynamic therapy as an adjunct for management of deep caries lesions - Study protocol for a randomized, parallel groups, controlled clinical trial
Trial registration {2a and 2b}.	This protocol is registered at ClinicalTrials.gov under the number NCT05236205. The register used for registration collects all items from the World Health Organization Trial Registration Data Set.
Protocol version {3}	This is the first version and it was first posted on 01/21/2022 and last updated on 05/10/2022, https://clinicaltrials.gov/ct2/show/NCT05236205?term=NCT05236205&draw=2&rank=1.
Funding {4}	One of the authors has received a grant from Conselho Nacional de Desenvolvimento Científico e Tecnológico (CNPq), under the number 306577/2020-8.
Author details {5a}	Post Graduation Program in Biophotonics Applied to Health Sciences, Universidade Nove de Julho, São Paulo, SP, Brazil; Paulo Picanço School of Dentistry, Fortaleza, CE, Brazil; Postgraduation Program in Health and Environment, Universidade Metropolitana de Santos, Santos, SP, Brazil.
Name and contact information for the trial sponsor {5b}	Postgraduate Program on Biophotonics Applied to Health Sciences, Universidade Nove de Julho, Vergueiro Street, 235/249 – Liberdade - ZIP 01504-001 - São Paulo- SP- Brazil. Phone No.: +55 11 3385-9088.
Role of sponsor {5c}	Collection, management, analysis, and interpretation of data; writing of the report; and the decision to submit the report for publication.

## Introduction

### Background and rationale {6a}

Antimicrobial photodynamic therapy (aPDT) is a promising new approach that aims to promote the reduction of microorganisms and it can be an innovative treatment to control oral biofilm microorganisms [1, 2]. Among these microorganisms, *Streptococcus mutans (SM)* and lactobacilli are usually present [1, 2]. Furthermore, it has been used in the treatment of dental caries [3]. Many studies have been conducted to confirm the effectiveness of this treatment modality [3–6].

The minimally invasive clinical treatment is considered an approach to the treatment of tooth decay, which acts on detection, diagnosis, interception, and treatment at the microscopic level. The partial removal of dental caries in order to maintain the integrity of the pulp is currently considered the treatment of choice for deep carious lesions, provided that some diagnostic principles are respected [7].

Currently, the treatment of these lesions is done with the removal of carious tissue using burs, hand excavators, or other techniques. Alternatively, chemo-mechanical caries removal agents can be used instead [8]. Papacarie ™ is considered a minimally invasive clinical treatment approach to treating dental caries with proven efficacy [3, 7–10]. It is a papain-based gel employing natural enzymes extracted from papaya husk that promotes the softening of the dentin. The gel acts upon the contaminated dentin and has an effect on local bacteria, as well as being biocompatible and having neutral pH [7]. However, it is necessary to improve its antimicrobial action. Clinical studies conducted to evaluate the antimicrobial potential of Papacarie™ (Fórmula e Ação, São Paulo, SP, Brazil) attribute its efficacy to its physical-chemical properties [8]. Partial caries removal by the Papacarie™ is capable of effectively dissolving the structure of caries-infected dentin through cysteine protease enzymatic action with additional bactericidal and anti-inflammatory properties [10].

This approach has been increasing in dentistry, being associated with antimicrobial photodynamic therapy (aPDT) [4–6]. The use of red/yellowish dyes is being researched for their application in aPDT. *Bixa orellana* is a plant native to Brazil. From its seeds, it is possible to extract a dye, popularly known as “urucum.” This dye has important antioxidant and antimicrobial activities, and recent studies have shown its potential as a photosensitizer in aPDT [3, 6, 11, 12]. Due to its positive characteristics, especially regarding the lack of mutagenic and cytotoxic activity and antimicrobial effect, the *Bixa orellana* extract (Fórmula e Ação, São Paulo, SP, Brazil) could be considered a suitable candidate as a photosensitizer. Using a dye with these characteristics would make aPDT more accessible [6, 13].

There are no controlled clinical trials in the literature that confirm the effectiveness of this type of therapy on deep caries-affected dentin associating Papacarie™ and aPDT with *Bixa orellana* extract, especially in the deep caries lesion. The justification for performing the treatment with Papacarie™ associated with aPDT using the *Bixa orellana* extract is to reduce the risk of pulp exposure in permanent teeth and increase the antimicrobial activity.

### Objectives {7}

#### General objectives

To investigate the use of Papacarie™ and aPDT with *Bixa orellana* extract on the caries-affected dentin of permanent teeth through a controlled clinical trial.

#### Specific objectives

- To investigate the efficacy of photodynamic therapy with *Bixa orellana* and Papacarie™ with regard to microbiological (counting of colony-forming units before and after treatments – CFU), radiographic (integrity of the periapical area and eventual changes in the radiolucent zones), and clinical aspects (retention of the restorative material in the cavity and occurrence of secondary caries) (primary objective);
- To evaluate the antimicrobial effect of aPDT on caries-affected dentin in permanent teeth (secondary objective);
- To perform a radiographic evaluation of the remaining dentin and radiographic density at 6 evaluation times, over a 12-month period (secondary objective); and
- To evaluate the need for local anesthesia during intervention and the degree of pain/discomfort (with a face scale) of children during the different procedures (secondary objective).

#### Hypothesis

Hypothesis (H0): The administration of Papacarie™ and aPDT with *Bixa orellana* extract is not effective in the treatment of caries-affected dentin in permanent teeth.

### Trial design {8}

**Fig. 1:**
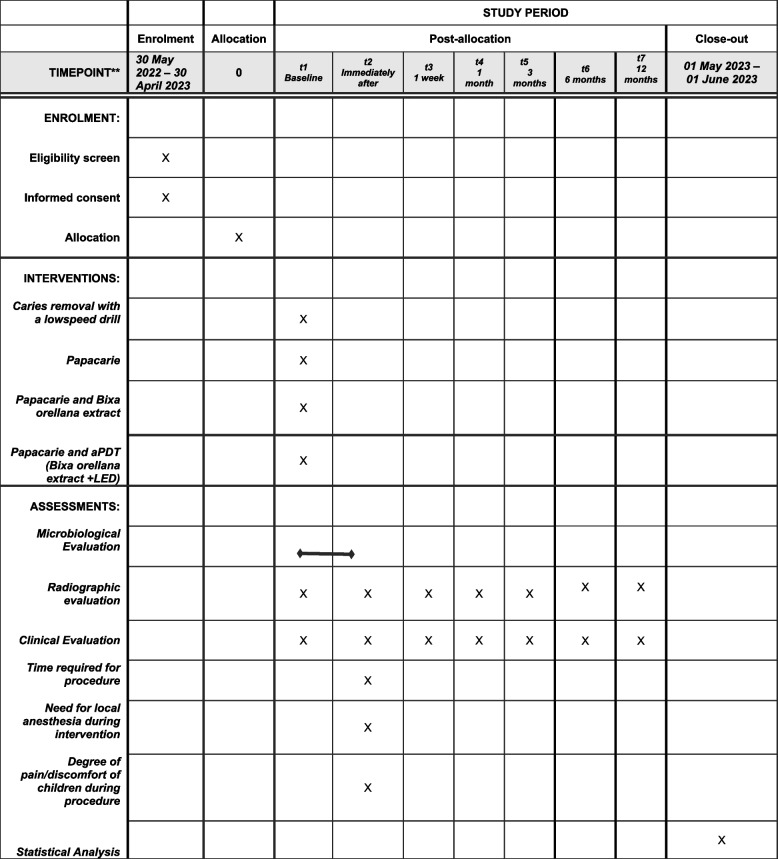
SPIRIT figure as recommended by the 2013 SPIRIT Statement, containing the description of procedures, interventions, assessments, and the study timeline

This is a study protocol for a randomized (1:1), single-blind (outcome assessors, only), parallel groups, equivalence, one-center, phase 2, controlled clinical trial. Figure 1 shows a schematic diagram and is the Standard Protocol Items: Recommendations for Interventional Trials (SPIRIT) figure.

## Methods: participants, interventions, and outcomes

### Study setting {9}

Participants will be selected from male and female children (with no restrictions regarding race or ethnicity) enrolled for treatment at the pediatric clinic of the dentistry course of Paulo Picanço School of Dentistry (Fortaleza-Ceará, Brazil).

### Eligibility criteria {10}

#### Inclusion criteria

- Adequate health with no systemic conditions (ASA 1);
- Adequate cooperation (scores 1 or 2 in Wright’s modification of Frankl’s behavior scale);
- Clinically presenting at least 1 permanent molar with an acute, active caries on the dentin not surpassing 2/3 and only involving the occlusal face, with direct view and access as well as no clinical or radiographic signs of pulp involvement. It is not intended to include more than one tooth per participant;
- Six to 12 years of age;
- Scores 3 (localized enamel breakdown due to caries with no visible dentine), 4 (underlying dark shadow from dentine), or 5 (distinct cavity with visible dentine) of the International Caries Detection and Assessment System criteria (ICDAS), in the occlusal surface; and
- The setting to access these criteria will be during a clinical and radiographic examination, performed in the pediatric clinic of the dentistry course of Paulo Picanço School of Dentistry.

#### Exclusion criteria

- Systemic adverse health condition;
- Uncooperative behavior;
- Clinically: caries involving enamel, deficient restorations, small carious lesions on dentin with no access for manual scalers, hidden caries, sign or symptom of pulp involvement, clinical impossibility of restoration; and
- Radiographically: evidence of pulp involvement, carious lesion extending beyond 2/3 of dentin.

### Who will take informed consent? {26a}

The same researcher (who is a dentist) responsible for the procedures will be in charge of approaching the guardians and obtaining the consent form. The researcher will explain all the procedures to be carried out, the purpose of the trial, the risks and benefits of participating in the research, and the fact that the volunteers have complete freedom to choose to participate and/or continue in the research, being able to withdraw at any time, without prejudice. The parents or guardians who agree to allow the minors they are responsible for to participate will sign a written statement of informed consent. They will be given enough time to consider and ask any questions. The children will sign a written assent term, with very simple, clear, and understandable language.

### Additional consent provisions for collection and use of participant data and biological specimens {26b}

Not applicable.

## Interventions

### Explanation for the choice of comparators {6b}

The use of a drill, which is the conventional and most commonly used method, will be compared with the use of Papacarie™ with or without the *Bixa orellana* extract, and with or without the irradiation for aPDT. This study will show which of these treatments will be more efficient with regard to microbiological, radiographic, and clinical aspects. The models, types, and details of all instruments have been detailed upon the first appearance in the text.

### Intervention description {11a}

*Group 1 –* Caries removal with a low-speed drill (control group):

1. Initial periapical radiography (Children's Insight IP-01 Periapical – Carestream, Rochester, Nova York, EUA);
2. Prophylaxis with a toothbrush and fluoride toothpaste (Colgate® Children Dr. Dentuço, Nova York, EUA) and fluoride toothpaste (Herjos Prophylactic Paste, Vigodent, Rio de Janeiro, Brazil);
3. Relative isolation with cotton roll (Dental Roller Cotton – Cremer, Santa Catarina, Brazil) and aspirator (Disposable Dental Sucker – SSPlus, Paraná, Brazil);
4. Microbiological sampling with ear curette (Meyhoefer auricular n° 2 curette (ABC Instrumentos Cirúrgicos, São Paulo, Brazil) for standardization of volume of carious tissue;
5. Removal of carious dentin with carbide burs (Carbide Drill No. 6 Spherical CA – Angelus, Paraná, Brazil) and manual instruments (Double Dentin Digger n° 17/18 - Millennium – Golgran, São Paulo, Brazil);
6. Second microbiological sampling with ear curette (Meyhoefer auricular n° 2 curette (ABC Instrumentos Cirúrgicos, São Paulo, Brazil) for standardization of volume of carious tissue;
7. Clinical inspection of texture of the remaining dentin with an exploratory probe (Eighth Exploration Probe – Golgran, São Paulo, Brazil), to check the absence of soft tissue;
8. Restoration with glass ionomer cement (Ketac Molar EasyMix – 3M ESPE); and
9. Clinical inspection for signs of infiltration and radiographic follow-up immediately, 1 week, and 1, 3, 6, and 12 months after treatment.

*Group 2 –* Partial removal of carious tissue with the administration of Papacarie™:

1. Initial periapical radiography;
2. Relative isolation with a cotton roll and aspirator;
3. Microbiological sample with otoscope curette to standardize the volume of carious tissue;
4. Application of Papacarie™ (Fórmula e Ação, São Paulo, SP, Brazil) for 5 min, removal of carious tissue around lateral walls of the cavity with a noncutting curette and no removal of carious tissue on pulp floor (partial removal) [10];
5. Washing of the cavity with water and drying with absorbent paper;
6. Second microbiological sampling;
7. Clinical inspection of texture of the remaining dentin with an exploratory probe, to check the absence of soft tissue;
8. Restoration with glass ionomer cement (Ketac Molar EasyMIx 3M ESPE); and
9. Clinical inspection for signs of infiltration and radiographic follow-up immediately, 1 week, and 1, 3, 6, and 12 months after treatment.

*Group 3 –* Partial removal of carious tissue with the administration of Papacarie™ and application of *Bixa orellana* extract (20% - seed extract diluted in mineral oil):

1. Initial periapical radiography;
2. Relative isolation with a cotton roll and aspirator;
3. Microbiological sample with otoscope curette to standardize the volume of carious tissue;
4. Application of Papacarie™ with *Bixa orellana* extract (20%) (Fórmula e Ação, São Paulo, SP, Brazil) for 5 min, removal of carious tissue around lateral walls of the cavity with a noncutting curette and no removal of carious tissue on pulp floor (partial removal) [10];
5. Washing of the cavity with water and drying with absorbent paper;
6. Second microbiological sample of remaining dentin with curette;
7. Clinical inspection of texture of the remaining dentin with an exploratory probe, to check the absence of soft tissue;
8. Restoration with glass ionomer cement (Ketac Molar EasyMIx 3M ESPE); and
9. Clinical inspection for signs of infiltration and radiographic follow-up immediately, 1 week, and 1, 3, 6, and 12 months after treatment.

*Group 4 –* Partial removal of carious tissue with the administration of Papacarie™, application of *Bixa orellana* extract (20%) and LED (aPDT):

1. Initial periapical radiography;
2. Relative isolation with a cotton roll and aspirator;
3. Microbiological sample with otoscope curette to standardize the volume of carious tissue;
4. Application of Papacarie™ with *Bixa orellana* extract (20%) for 5 min and the light-emitting diode (LED) light curing device (Valo Cordless Ultradent®, South Jordan, UT, USA) an office appliance, with a coupled radiometer, and a spectrum of 440–480 nm will be used. Both the volunteer to be treated and the professional will be using specific eye protection glasses. The active end of the LED will be coated with clear disposable plastic (PVC), thus avoiding cross-contamination. Removal of carious tissue around lateral walls of the cavity with a noncutting curette and no removal of carious tissue on pulp floor (partial removal) [10–12];
5. Irradiation of dental tissue for 1 min on a single point;
6. Washing of the cavity with water and drying with absorbent paper;
7. Second microbiological sample of remaining dentin with curette;
8. Clinical inspection of texture of the remaining dentin with an exploratory probe, to check the absence of soft tissue;
9. Restoration with glass ionomer cement (Ketac Molar EasyMIx 3M ESPE); and
10. Clinical inspection for signs of infiltration and radiographic follow-up immediately, 1 week, and 1, 3, 6, and 12 months after treatment.

### Criteria for discontinuing or modifying allocated interventions {11b}

If the participant shows signs/symptoms of pulpal involvement and requires other treatment modalities, treatment will be discontinued and the participant data will be excluded from the study. However, they will still be followed up for safety assessment.

### Strategies to improve adherence to interventions {11c}

Participants will be enrolled for treatment at the pediatric clinic of the dentistry course of Paulo Picanço School of Dentistry (Fortaleza-Ceará, Brazil). As they will already be in treatment, we believe this will improve adherence. They will also be told about the importance of the follow-up to complete the research data.

### Relevant concomitant care permitted or prohibited during the trial {11d}

Children involved in the study may present caries involving enamel, deficient restorations, small carious lesions on dentin with no access for manual scalers, hidden caries, sign or symptom of pulp involvement, clinical impossibility of restoration, evidence of pulp involvement, or carious lesion extending beyond 2/3 of dentin in other teeth, which are not involved in the study. In these cases, the researchers will provide the necessary care for these other conditions.

### Provisions for post-trial care {30}

No major harms are expected, but possible intercorrences, such as tooth pain, will be monitored and recorded. Any additional assistance participants may need, will be provided.

### Outcomes {12}

For the treatment of caries lesions to be considered successful, it should be able to remove infected tissue and microorganisms in a sufficient amount, so that, in the control session, the teeth that were once infected do not show any signs of remaining infected tissue beneath the restorative material. Consequently, the microbiological evaluation had been chosen as the primary outcome. However, factors such as the radiological exam and the clinical aspects are also important for the control section, to show the presence or lack of success. Therefore, the microbiological, radiographic, and clinical evaluations should be considered as primary outcomes.

#### Microbiological evaluation

This is one of the primary outcomes of the study. A sample of caries-affected dentin will be taken from each selected tooth before the removal of the carious tissue. The samples will be standardized with the use of a Meyhoefer auricular curette n° 2 (ABC Instrumentos Cirúrgicos, São Paulo, Brazil), because it has a hole in its tip, and placed into test tubes containing 3.8 ml of the transport medium (Phosphate Buffered Saline (PBS) - Thermo Fisher Scientific, Massachusetts, EUA). The dental tissue will be dispersed in the transport tube (Microtubo Tipo Eppendorf Cap. 1.5 ml – GlobalPastic, São Paulo, Brazil) containing glass pearls through agitation at maximum speed in a vortex device (Agitador Para Tubos Vortex – Kasvi, Paraná, Brazil) for 30 s to homogenize the biological material. The biofilm will be diluted in series on the order of 10^1^ to 10^6^ in peptone water and inoculated in culture media in Petri dishes (Placa de Petri Descartável Estéril - PRO-LAB MATERIAIS PARA LABORATORIOS LTDA, São Paulo, Brazil). Aliquots of dilutions 10^4^, 10^5^, and 10^6^ will be sewn on the surface of Brucella agar (Difco Laboratories, Detroit, Michigan) containing defibrinated sheep blood (50ml/L), hemin (5 mg/ml), and menadione (10mg/ml) for the determination of the total number of viable microorganisms (VM). Aliquots of dilutions 10^3^ and 10^4^ will be sewn on Mitis Salivarius agar (Difco Laboratories, Detroit, Michigan) for the determination of the total number of streptococcus (S). Aliquots of dilutions 10^1^ and 10^2^ will be sewn on Mitis Salivarius agar with the addition of debacitracin for the determination of the population of streptococcus of the mutans group (SM).

Aliquots (100 ml) from each dilution will be sewn onto the surface of agar and spread with the aid of a Drigalski spatula. Undiluted aliquots and aliquots from dilution 10^2^ (100 ml) will be pour-plated on Rogosa SL agar (Difco Laboratories, Detroit, Michigan) for the determination of lactobacilli (LB). The Brucella agar dishes will be incubated in an anaerobic chamber (PLAS by LABS, Lansing, MI) at 37°C for 7 days. The Mitis Salivarius agar and Mitis Salivarius Bacitracin dishes will be incubated in a 10% CO_2_ atmosphere (CO_2_ greenhouse, Shel Lab, mod. 2123, Oregon) at 37°C for 48 h. The dishes containing Rogosa agar will be incubated in a 10% CO_2_ atmosphere at 37°C for 72 h. After incubation, the characteristic colonies in each dish will be counted with the aid of a stereomicroscope at a magnification of 10 times in dilutions with 30 to 300 colonies per dish. All procedures will be performed in duplicate, and the mean of the counts will be calculated. The results will be expressed in colony-forming units (CFU) of SM and LB as well as in proportion of streptococcus (% S/VM), SM group (% SM/VM and lactobacilli (% LB/VM) in relation to the total of viable microorganisms (VM). For SM, the proportion in relation to the total of streptococci (% SM/S) will also be calculated. Immediately after the removal of the carious tissue, samples of the remaining dentin will be taken with a Meyhoefer auricular n° 2 curette and the procedures will be repeated [14].

#### Radiographic evaluation

This is one of the primary outcomes of the study. The radiographic examinations will be carried out in the following manner: (1) periapical radiographs will be used as a diagnostic aid; (2) after the incomplete removal of the demineralized dentine and the temporary filling of cavity, a bitewing radiograph it will be taken to allow the analysis of the radiolucent zone (RZ), that is, the amount of demineralized dentine left; (3) immediately, 1 week, and 1, 3, 6 and 12 months, periapical and bitewing radiographs will be taken to analyze the integrity of the periapical area and the eventual changes in the RZ.

To obtain geometric standardization of films, bitewing film holders will be used. The images obtained before and after the treatment will be digitized by a ScanJet 6100 scanner Hewllet-Packard (OR, USA). The images will be stored in maximum-quality JPG format. The Imagelab software (version 2.3, SoftiumSistemas de Informatica, São Paulo, Brazil) will be used to analyze the images (equalize and subtract). Change in the density measurement of the RZ during treatment will be evaluated blind through digital subtraction of radiographic images in relation to the initial exams in the experimental periods (immediately, 1 week, and 1, 3, 6, and 12 months).

The radiographic subtraction process will consist of comparing two images. By definition, a digital subtraction image from a site where no change in density has occurred would show a complete cancelation of all anatomical structures, eliminating the constant structures present in both. Any change in the structures indicates a change in density. This method requires standardized radiographs regarding the X-ray projection, exposure time, film, and development. Nonetheless, the procedures will be standardized; radiographs will be performed at different moments, which causes them to have different densities. To correct for any changes in density, the gray-level histograms of the two images will be compared and adjusted using a nonparametric contrast correction algorithm. The image radiograph equalization will be done in the following way: the radiograph taken immediately after the treatment and the one taken after each period of treatment (immediately, 1 week, and 1, 3, 6, and 12 months) will be placed beside one another on the computer screen. The radiograph that shows the best distribution of gray tonalities on the histogram will be chosen as a model, and the other image will be equalized according to the model.

The subtraction will be carried out the following way: the radiograph taken immediately after the treatment and the images obtained after each of the experimental periods will be overlapped. The anatomic details of the two images will be matched by rotation or moving the images horizontally or vertically. The radiolucent zone beneath the restoration and two control areas (CA1 and CA2) will be selected. The control areas will be dentinal areas (mesial and distal) close to the RZ. The difference in radiographic density between the two radiographs will be determined for each selected area using a gray tonalities scale. There are 256 individual shades of gray in an 8-bit pixel going from 0 (black) to 255 (white). This is the pixel’s grayscale resolution. An average gray level value of 128 (the middle of the digitizer gray level range set by the software) would show up at each pixel. Areas with gray levels <128 in the subtraction image would indicate a loss in density and gray levels >128 would indicate an increase in density [15].

#### Evaluation of time required for the procedure (secondary outcome)

The time required for each procedure will be measured using a digital stopwatch (Kenko, Hong Kong) in minutes and seconds from the onset of treatment until the complete removal of the carious tissue. The time will be recorded on a specific chart for analysis. The need or non-need for anesthesia will also be recorded.

#### Evaluation of the need for local anesthesia during intervention and degree of pain/discomfort of children during the procedure (secondary outcome)

All interventions will be initiated without the prior administration of local anesthesia. The children will be told that anesthesia could be administered at any time during the intervention. A face scale with different expressions will be used to evaluate the need for local anesthesia and the child will be asked to point to the expression that most corresponds to his/her degree of pain/discomfort.

Interpretation of face scale:

1. No pain.
2. Mild pain.
3. Moderate pain.
4. A little worse pain.
5. Strong pain.
6. Worst pain.

#### Clinical evaluation

This is one of the primary outcomes of the study. The clinical evaluation will be performed by a researcher blinded to the different treatment groups. The criteria used for the evaluation will be the retention of the restorative material in the cavity and the occurrence of secondary caries. The evaluation scores will be based on the criteria below. Digital photographs of the restorations will also be taken and serve to complement the clinical and radiographic findings. The visual demonstration will contribute to any necessary clarifications and facilitate the discussion and documentation of the cases. Thus, digital photographs will be taken of all teeth in the different groups before and after the interventions. We believe participants will be present for the follow-ups due to concerns and other possible treatments in the clinic.

- 0 = present; no defects;
- 1 = present; small marginal defects measuring less than 0.5 mm in depth; no need for repair;
- 2 = present; small marginal defects measuring 0.5 mm to 1mm in depth; need for repair;
- 3 = present; large marginal defects measuring 1 or more mm in depth; need for repair;
- 4 = absent; restoration nearly or completely lost; need for treatment;
- 5 = absent; additional treatment having been performed for some reason;
- 6 = tooth absent for any reason;
- 7 = present; surface wear measuring less than 0.5 mm in depth; no need for replacement;
- 8 = present; surface wear greater than 0.5 mm in depth; need for replacement; and
- 9 = impossible to diagnose.

### Participant timeline {13}

The participant timeline is shown in Fig. 1.

### Sample size {14}

**Fig. 2:**
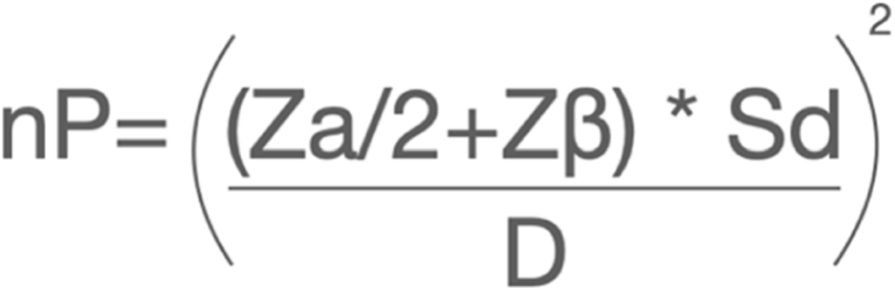
Sample size calculation used for this clinical research

**Fig. 3:**
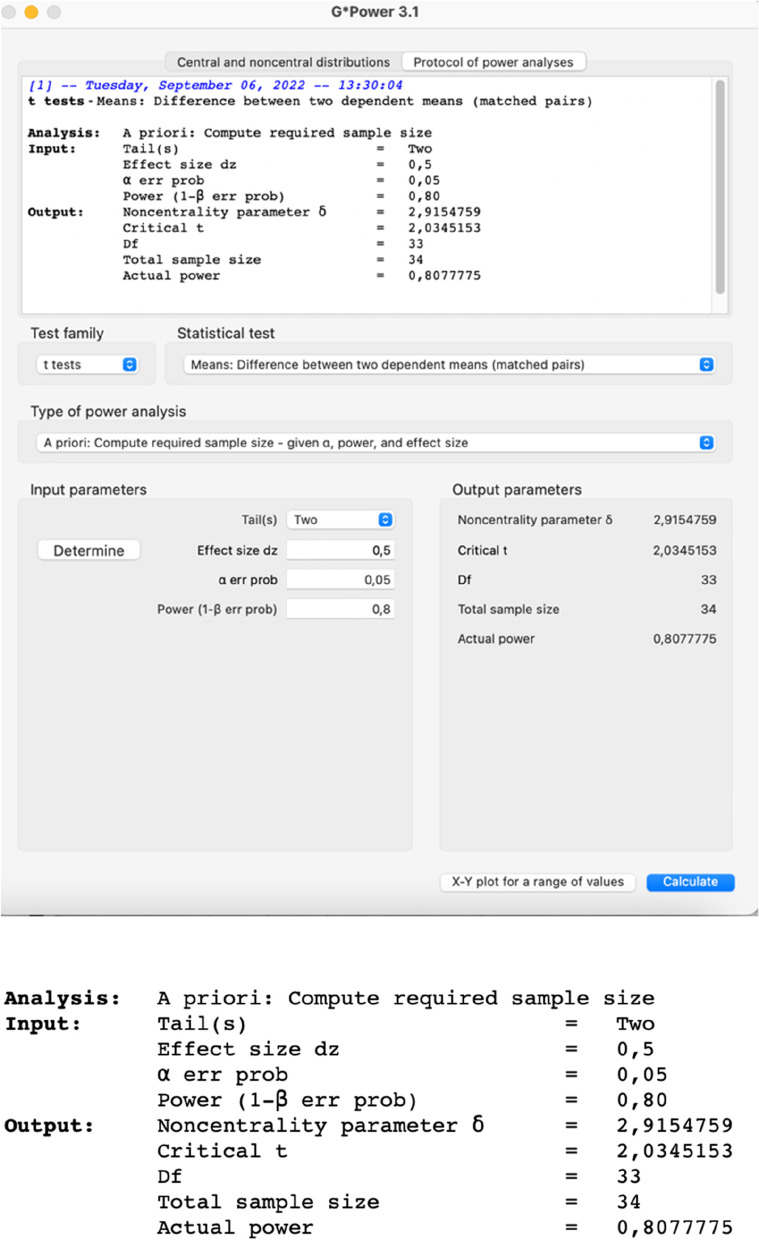
Determination of the sample size for this clinical research in G*Power 3.1

The sample size was calculated based on a previous study in the literature [16, 17] (Fig. 2), considering the expected difference and standard deviation and weighting colony-forming units (CFU) of bacteria. For the statistical calculation, paired samples were considered, with α=5% and an 80% test power. The minimum number in this clinical trial was determined to be 34 teeth per group. Therefore, 40 teeth per group will be recruited to compensate for possible dropouts during the experimental period. G*Power 3.1 was used to perform the calculations (Fig. 3).

### Recruitment {15}

As participants will already be in pediatric dentistry treatment, we believe recruitment will be feasible. Participants will be approached at their usual clinic appointment. We believe participants will be present for the follow-ups due to concerns and other possible treatments in the clinic.

## Assignment of interventions: allocation

### Sequence generation {16a}

Block randomization will be performed using the website randomizer.org, which will generate 4 sets of numbers, according to the number of research groups and participants. Opaque envelopes will then be ordered, with the corresponding groups written inside, and kept in a locked room inside the clinic, accessible only to the researchers. A researcher who will not be involved in the data collection will be responsible for generating the sequence and making and sealing the envelopes. The participant will remove the envelope at the time of treatment.

### Concealment mechanism {16b}

Numbers according to treatments will be kept in opaque envelopes:

- Number 1 – Traditional caries removal with a low-speed drill (Group 1);
- Number 2 – Partial removal of carious tissue with the administration of Papacarie™ (Group 2);
- Number 3 – Partial removal of carious tissue with the administration of Papacarie™ and application of *Bixa orellana* extract (20%) (Group 3); and
- Number 4 – Partial removal of carious tissue with the administration of Papacarie™, application of *Bixa orellana* extract (20%), and LED (aPDT) (Group 4).

### Implementation {16c}

The researchers responsible for the treatment application will enroll participants and assign participants to interventions, only the outcome assessors will be blind. The allocation sequence will be made by a different researcher, who will not be involved in the procedures.

## Assignment of interventions: blinding

### Who will be blinded {17a}

The clinical evaluations of the carious tissue removal, as well as the microbiological and radiographic analyses, will be performed by examiners blinded to the treatments performed on each tooth. Only the researcher in charge of procedures will know to which group the participants belong.

### Procedure for unblinding if needed {17b}

As the researchers responsible for clinical care are aware of the participant allocation, unblinding of the assessors would not be required.

## Data collection and management

### Plans for assessment and collection of outcomes {18a}

Researchers will be previously trained to collect data and perform evaluations, according to the parameters described in outcome measures. The previously trained examiner will evaluate all cavities after the respective interventions and will test the hardness of the remaining dentin. For carious tissue to be considered removed, there must be an agreement between the operator and the examiner. To determine interexaminer agreement regarding the clinical and radiographic evaluations by examiners, the calculation of Kappa coefficients will be used at baseline and follow-up appointments. Interexaminer agreement must be greater than 85% (*K*>0.85) Firstly, the researchers responsible for the procedures will write the information in paper case report forms. Then, the data will be entered electronically in Excel datasets, in computers to which only the authors will have access.

### Plans to promote participant retention and complete follow-up {18b}

As participants will already be in pediatric dentistry treatment, we believe recruitment will be feasible. We believe participants will be present for the follow-ups due to concerns and other possible treatments in the clinic. Phone texts and calls will also be used as visit reminders.

### Data management {19}

All data will be entered electronically, in Excel files, and stored in computers and hard discs. The participants’ files will be accessible only to the authors of this study and only they will be able to edit the information, maintaining data accuracy and validation.

### Confidentiality {27}

The data sets generated and analyzed during the study will be available from the corresponding author at reasonable request. Once the data is entered electronically, participants’ identification details will no longer be attached to their data, they will be reported only by codes. After the analysis of the data, volunteers will be invited to a meeting and the results will be shared, in case they wish to attend it. The authors also intend to publish the results.

### Plans for collection, laboratory evaluation, and storage of biological specimens for genetic or molecular analysis in this trial/future use {33}

Not applicable.

## Statistical methods

### Statistical methods for primary and secondary outcomes {20a}

The data will be stored in Excel datasets and analyzed statistically by employing different tests and considering a 5% level of significance. Only the researchers involved in the study will have access to the data, even if the study is discontinued. The results will be submitted to the Shapiro-Wilk test to check for normality. In case data distribution is normal, analysis of variance (ANOVA) will be used for both the dependent and independent variables. In case the data distribution is not normal, the Friedman test will be used for the dependent variables, followed by the Wilcoxon test as post hoc. For independent variables, the Kruskal-Wallis test will be used, followed by the Mann-Whitney test as post hoc. Pearson (for normal data) or Spearman (for non-parametric data) correlation coefficients may be calculated to determine correlations among the variables.

### Interim analyses {21b}

The corresponding author makes the final decision to terminate the trial, if necessary. This will only happen in case it becomes impossible to clinically attend the participants as, for example, in outbreaks of infectious diseases. The authors will have access to these interim results.

### Methods for additional analyses (e.g., subgroup analyses) {20b}

Not applicable.

### Methods in analysis to handle protocol non-adherence and any statistical methods to handle missing data {20c}

No losses greater them 5% of the sample are expected, and if they happen, pairwise deletion will be used.

### Plans to give access to the full protocol, participant-level data and statistical code {31c}

The final datasets will be available from the corresponding author, at a reasonable request. The authors have the intention of publishing the results, in articles and conferences.

## Oversight and monitoring

### Composition of the coordinating center and trial steering committee {5d}

The authors themselves will coordinate and steer the trial.

### Composition of the data monitoring committee, its role and reporting structure {21a}

Seeing as this is not a study that involves significant safety concerns, risks, or complexity, nor is it a multicenter study of long duration, no data monitoring committee was considered required.

### Adverse event reporting and harms {22}

No major harms are expected, but possible intercorrences, such as tooth pain, will be monitored and recorded. Any additional assistance participants may need, will be provided.

### Frequency and plans for auditing trial conduct {23}

Monitoring will be carried out by sponsor personnel or representatives at the investigation site, monthly.

### Plans for communicating important protocol amendments to relevant parties (e.g. trial participants, ethical committees) {25}

Changes in the protocol will be reported to the Ethics Committee of Faculdade Paulo Picanço and altered in ClinicalTrials.gov.

### Dissemination plans {31a}

After the analysis of the data, volunteers will be invited to a meeting and the results will be shared, in case they wish to attend it. The authors also intend to publish the results.

## Discussion

Studies have shown that the isolated action of aPDT is not completely efficient for the microbial reduction and painful symptoms of patients [18], highlighting the importance of dental professionals in both the treatment and prevention of deep caries-affected dentin of permanent teeth [19]. Procedures using aPDT have also been developed to the treatment of dental caries [3, 4, 19]; however, there are few controlled clinical trials in the literature confirming its efficacy. Papacarie™ is a gel composed of papain and chloramine [8, 9] employed for the partial removal of carious tissue, effective against bacteria due to its properties, but some studies show that the antibacterial action is not so evident. *Bixa orellana* is a Brazilian plant, and its seeds produce one of the most frequently used worldwide dyes, which is called annatto or “urucum” [6, 20]. Its leaves and seeds were reported to have antimicrobial, antifungal, anticonvulsant, analgesic, and anti-inflammatory activities, and showed important activity against Gram-positive and Gram-negative bacteria [21, 22]. The *Bixa orellana* extract proposed with this study could enhance the antimicrobial action through aPDT [6] and improve the effectiveness of conservative treatments using the selective removal of carious tissue and the use of Papacarie™. In addition, there is a tendency for patients to value new treatment technologies, which can increase their satisfaction with this therapy.

## Trial status

This protocol is registered at ClinicalTrials.gov under the number NCT05236205 and it was first posted on 01/21/2022 and last updated on 05/10/2022, https://clinicaltrials.gov/ct2/show/NCT05236205?term=NCT05236205&draw=2&rank=1. Recruitment will take place from 30 May 2022 to 30 April 2023.

## Data Availability

The data sets generated and analyzed during the study will be available from the corresponding author at reasonable request. After the analysis of the data, volunteers will be invited to a meeting and the results will be shared, in case they wish to attend it. The authors also intend to publish the results.

## Abbreviations

aPDT: Antimicrobial photodynamic therapy
CFU: Colony-forming units
LB: Lactobacilli
LED: Light-emitting diode
S: *Streptococcus*
SM: *Streptococcus mutans*
SPIRIT: Standard Protocol Items: Recommendations for Interventional Trials
VM: Viable microorganisms
